# Supplementary material for: Role and mechanism of NCAPD3 in promoting malignant behaviors in gastric cancer
Source: Front Pharmacol. 2024 Apr 22;15:1341039. doi: 10.3389/fphar.2024.1341039 (PMC11070777; doi:10.3389/fphar.2024.1341039)
Supplement: Supplementary file 11 [file DataSheet2.ZIP › GSEA/Oncogenic signature/my_analysis.Gsea.1599461962066/EIF4E_DN.html]

Details for gene set EIF4E\_DN[GSEA]

|  || Dataset | filtered\_dataset.sample\_info.cls#WT\_versus\_NCAPD3\_MUT |
| Phenotype | sample\_info.cls#WT\_versus\_NCAPD3\_MUT |
| Upregulated in class | WT |
| GeneSet | EIF4E\_DN |
| Enrichment Score (ES) | 0.28524378 |
| Normalized Enrichment Score (NES) | 1.0892028 |
| Nominal p-value | 0.34879726 |
| FDR q-value | 0.65099025 |
| FWER p-Value | 1.0 |
Table: GSEA Results Summary

  

Fig 1: Enrichment plot: EIF4E\_DN      
 Profile of the Running ES Score & Positions of GeneSet Members on the Rank Ordered List

  

| SYMBOL | TITLE | RANK IN GENE LIST | RANK METRIC SCORE | RUNNING ES | CORE ENRICHMENT || 1 | 10513 | APPBP2 | 28 | 1.000 | 0.0862 | Yes |
| 2 | 23054 | NCOA6 | 77 | 0.838 | 0.1408 | Yes |
| 3 | 7976 | FZD3 | 88 | 0.815 | 0.2202 | Yes |
| 4 | 9897 | KIAA0196 | 197 | 0.682 | 0.2152 | Yes |
| 5 | 23001 | WDFY3 | 240 | 0.642 | 0.2533 | Yes |
| 6 | 54477 | PLEKHA5 | 286 | 0.605 | 0.2852 | Yes |
| 7 | 9743 | ARHGAP32 | 413 | 0.528 | 0.2511 | No |
| 8 | 1793 | DOCK1 | 489 | 0.480 | 0.2483 | No |
| 9 | 57496 | MKL2 | 658 | 0.398 | 0.1702 | No |
| 10 | 4008 | LMO7 | 909 | -0.335 | 0.0267 | No |
| 11 | 10724 | MGEA5 | 983 | -0.394 | 0.0163 | No |
| 12 | 1604 | CD55 | 1090 | -0.468 | -0.0100 | No |
| 13 | 4651 | MYO10 | 1213 | -0.588 | -0.0349 | No |
| 14 | 195814 | SDR16C5 | 1314 | -0.728 | -0.0292 | No |
| 15 | 6840 | SVIL | 1377 | -0.916 | 0.0236 | No |
Table: GSEA details [plain text format]

  

Fig 2: EIF4E\_DN      
 Blue-Pink O' Gram in the Space of the Analyzed GeneSet

  

Fig 3: EIF4E\_DN: Random ES distribution      
 Gene set null distribution of ES for **EIF4E\_DN**
